# Salivary Extracellular Vesicles: Biomarkers and Beyond in Human Diseases

**DOI:** 10.3390/ijms242417328

**Published:** 2023-12-10

**Authors:** Jialing Wu, Gege Liu, Rong Jia, Jihua Guo

**Affiliations:** 1 State Key Laboratory of Oral & Maxillofacial Reconstruction and Regeneration, Key Laboratory of Oral Biomedicine Ministry of Education, Hubei Key Laboratory of Stomatology, School & Hospital of Stomatology, Wuhan University, Wuhan 430072, China; jialingwu@whu.edu.cn (J.W.); wenge_liu@whu.edu.cn (G.L.); jiarong@whu.edu.cn (R.J.); 2 Department of Endodontics, School & Hospital of Stomatology, Wuhan University, Wuhan 430079, China

**Keywords:** saliva, extracellular vesicles, biomarker, oral diseases

## Abstract

Extracellular vesicles, as bioactive molecules, have been extensively studied. There are abundant studies in the literature on their biogenesis, secretion, structure, and content, and their roles in pathophysiological processes. Extracellular vesicles have been reviewed as biomarkers for use in diagnostic tools. Saliva contains many extracellular vesicles, and compared with other body fluids, it is easier to obtain in a non-invasive way, making its acquisition more easily accepted by patients. In recent years, there have been numerous new studies investigating the role of salivary extracellular vesicles as biomarkers. These studies have significant implications for future clinical diagnosis. Therefore, in this paper, we summarize and review the potential applications of salivary extracellular vesicles as biomarkers, and we also describe their other functions (e.g., hemostasis, innate immune defense) in both oral and non-oral diseases.

## 1. Introduction

Extracellular vesicles (EVs) are particles secreted by cells into the extracellular environment [1,2] which are enveloped by the lipid bilayer and contain donor-cell-derived proteins and cytoplasmic and genetic materials [1]. EVs can be mainly divided into exosomes, microvesicles (MVs), apoptotic bodies, and other subtypes according to their size, morphology, biosynthesis, and secretion methods [2,3]. The circulating particles are mainly exosomes and MVs [4], which contribute to intercellular communication and have attracted much attention. In the past, it was thought that the secretion of EVs was due to donor cells discarding unwanted components; however, recent studies suggest that the release of EVs is involved in the physiological activity of intercellular communication and plays a pivotal role in information transfer [5]. Intercellular communication methods are varied; they include direct cell-to-cell interaction, secreting soluble factors (e.g., growth factors, hormones), and communication through EVs [2,6]. The reason why EVs are unique in intercellular communication is that they can carry many biologically active molecules, such as nucleic acids, proteins, and lipids [2], and they can mediate the physiological activity of the recipient cells [2,6,7]. Additionally, EVs show promise for application in clinical settings, mainly because they are rich in the biomarkers of donor cells [3] and widely distributed in body fluids, making them easy to collect for use in diagnosing disease, delivering drugs, and observing therapeutic responses [5,8,9]. Currently, the methods for isolation and purification of EV subclasses are not well established [5] and it is not yet possible to completely separate exosomes from MVs [4,9]. Further development is needed to meet the demands for EVs as biomarkers, vaccines, drug delivery devices, and therapeutic tools [4].

EVs are abundant in saliva [10,11]. Molecular cargoes in salivary extracellular vesicles (sEVs) will change depending on the pathophysiological state of the donor cells [12,13], suggesting that these molecular cargoes can be potential biomarkers of diseases. Studies have reported that sEVs can be used in the early detection and monitoring of oral and systemic diseases [14,15]. Additionally, as a liquid biopsy for disease diagnosis, sEVs are non-invasive and cost-effective. Thus, sEVs have attracted much attention and become a rapidly advancing field of study [16].

In the past two years, there have been a large number of new sEV-related studies. This article, as an updated review, summarizes the literature from the most recent two years and previously, in order to discuss the characteristics of sEVs and their roles in human diseases.

## 2. Characteristics of sEVs

Saliva is a biological fluid produced by the three pairs of major salivary glands and many minor salivary glands in the oral cavity. When referring to the term “spit” in daily life, it is more appropriate to describe it as whole-mouth saliva (WMS) [17]. In addition to 99% water, WMS also contains organic substances (e.g., proteins, enzymes, lipids), inorganic ions (e.g., potassium, sodium, calcium), as well as epithelial cells, bacteria, gingival crevicular fluid, and secreted vesicles [16,18,19]. The composition of saliva can change due to oral diseases or systemic diseases, and it has been confirmed that salivary mRNA can be used for cancer monitoring [20]. Saliva is an optimal candidate for monitoring oral diseases, as it not only contains abundant microparticles, but it is easier to obtain compared with other body fluids such as blood, urine, and breast milk [18]. Furthermore, RNA enclosed in EVs can be protected from degradation by salivary nucleases and provide disease-related information, highlighting the advantages of sEVs as biomarkers. SEVs also participate in many physiological and pathological activities. Evidence has proved that salivary exosomes from periodontitis participate in the transmission of inflammatory signals, thereby promoting the process of periodontitis [21]. Moreover, experiments in vitro demonstrated that labeled saliva exosomes could be taken up by keratinocytes and transmit their genetic information (mRNA) to oral keratinocytes, thereby altering the gene expression [22]. Further, sEVs from mice bearing pancreatic tumors could inhibit NK cells’ cytotoxic potential against tumor cells, thus promoting tumor immunity escape [23]. In addition to serving as biomarkers and facilitating information communication, sEVs also have other functions, such as participating in innate immune defense [24] and coagulation [25], and serving as drug delivery system carriers [26].

### 2.1. Types of EV

**Figure 1:**
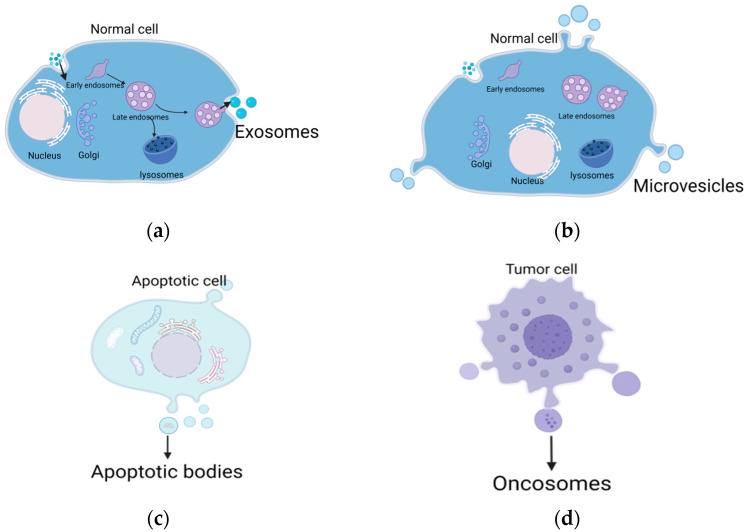
Different types of cells can secrete various types of extracellular vesicles. (**a**) The releasing process of exosomes, which rely on the endosomal system. (**b**) The release of MVs, which directly bud from the plasma membrane. (**c**) Cells undergoing programmed cell death can form apoptotic bodies. (**d**) Oncosomes are secreted by tumor cells, which have a larger diameter compared with other extracellular vesicles. Figures were created with BioRender.com (accessed on 17 October 2023).

EVs are a type of secreted vesicle carrying cargoes from their parental cells. The cargoes mainly consist of biologically active proteins, nucleic acids, and lipids [2]. Initially, it was believed that the release of EVs was merely a way for cells to discard unwanted waste. Now it is known that EVs play functional roles in both physiological and pathological processes, such as intercellular communication [2,19,27], coagulation [25], and tumor metastasis [2]. EVs can be categorized into exosomes, MVs, and apoptotic bodies based on their size, morphology, density, biogenesis pathways, and secretion methods [1,2,3] (Figure 1).

#### 2.1.1. Exosomes

The term “exosomes” was first used to describe vesicles released by different cells that contain 5′-nucleotidase activity [28]. In 1987, exosomes were defined as vesicles formed by the inward budding of multivesicular bodies (MVBs) during the differentiation of reticulocytes [29]. According to observations conducted via electron microscopy, the size of exosomes is approximately 30–100 nm, sometimes up to 150 nm [28]. Exosomes can be secreted by various kinds of cells [30,31] and are widely distributed in body fluids, including blood [29], synovial fluid [32], saliva [33,34], urine [35], breast milk [36], pleural effusion [37], and amniotic fluid [31]. The biogenesis of exosomes primarily relies on the endosomal system. First, the membrane of the endosome buds inward to form MVBs [4,38], which contain numerous intraluminal vesicles (ILVs) [1]. Then, a portion of the MVBs is transported to lysosomes for degradation [39], while another portion is transported to the plasma membrane. Finally, they fuse with the plasma membrane and release the ILVs, called exosomes [40,41].

#### 2.1.2. MVs

MVs, initially known as “platelet dust”, were believed to be products of activated platelets and reticulocytes, playing roles in clotting [41,42]. MVs can also be released by various kinds of cells, such as mesenchymal stem cells (MSCs) and adipose-derived stem cells (ADSCs) [43]. The diameter of MVs typically ranges from 50 to 1000 nm [38]. MVs secreted by tumor cells are also called oncosomes, which can reach 10 μm in diameter [44]. MVs are usually formed by direct budding or extrusion of the cell membrane [45,46].

#### 2.1.3. Apoptotic Bodies

Blebbing is a very common feature of apoptotic cells. Apoptotic bodies are released from apoptotic cells through a physical process mediated by actomyosin [47]. After the cell membrane separates from the cortical cytoskeleton, the apoptotic body is formed; it has a diameter of 1000–5000 nm [48]. Apoptotic bodies are filled with cellular content, such as intact organelles, condensed chromatin, and proteins [47,48,49].

### 2.2. Origins of sEVs

**Figure 2:**
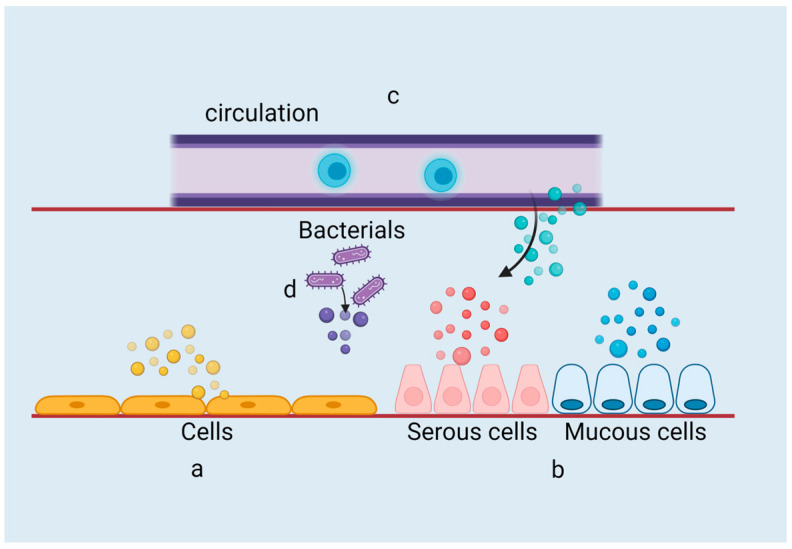
The origins of sEVs: (a) SEVs can be directly secreted from oral epithelial cells and granulocytes in the oral cavity. (b) They can also be secreted by the major salivary glands, including the parotid gland, submandibular gland, and sublingual gland, as well as many minor salivary glands into the oral cavity. (c) Some sEVs in the oral cavity can originate from circulation. These circulation-derived extracellular vesicles often contain extracellular proteins and secreted proteins. (d) The bacteria present in the oral cavity can also secrete EVs, known as bEVs, which play an important role in oral health. The different colors/ arrows represent different origins of sEVs. The figure was created with BioRender.com (accessed on 17 October 2023).

sEVs have various different origins and can enter into saliva through several pathways, such as originating from cells in the oral cavity, the salivary glands, or the circulation. Moreover, bacterial-derived extracellular vesicles also constitute a significant portion of sEVs (Figure 2).

#### 2.2.1. Cellular Origin

A portion of sEVs is directly released from cells. Epithelial cells and granulocytes in the oral cavity can directly release EVs into saliva, and most cell-derived sEVs are released from those cell types [20,22,25].

#### 2.2.2. Glandular Origin

Exosomes have been detected in “pure glandular saliva” secreted from the parotid gland and submandibular gland, indicating that parts of sEVs are derived from the salivary glands [50,51]. Moreover, exosomes secreted by individual glands are derived from cells within that particular gland, which means we may monitor the physiologic state of glands through exosomes [51].

#### 2.2.3. Circulatory Origin

Through analyzing salivary exosome proteins it was found that approximately 40% of these proteins are extracellular proteins or secretory proteins, suggesting that some of the vesicles in saliva originate from circulation [52]. By constructing a mouse model of pancreatic cancer, exosomes containing pancreatic-cancer-specific transcriptome biomarkers (mRNA) were detected in saliva, proving that tumor-derived exosomes may enter into saliva through circulation [53]. In addition, an earlier experiment found after injecting H460 human lung cancer cells which stably express hCD63-GFP into immunocompromised mice, exosome-like microvesicles (ELMs) expressing hCD63 were detected in mouse saliva, and species-specific CD63 protein and GAPDH mRNA were also detected. That is to say, ELMs can carry tumor-cell-specific mRNA into the circulation, and this eventually reaches the saliva [54]. The evidence not only suggests that salivary exosomes can originate from the circulation, but also provides a theoretical basis for salivary exosomes acting as disease biomarkers.

#### 2.2.4. Bacterial Origin

As mentioned above, almost all cells can secrete EVs, and even bacteria can release vesicles from their cell membranes. Since the oral cavity is a bacteria-rich environment, it is undeniable that saliva also contains a large number of EVs secreted by bacteria, which are of great significance in the study of oral microbiology and diseases.

EVs secreted by bacteria are collectively referred to as bacterial extracellular vesicles (bEVs). The vesicles secreted by Gram-positive bacteria are called cytoplasmic membrane vesicles (CMVs). These originate from the cytoplasmic membrane and carry components from the cytoplasm. Vesicles secreted by Gram-negative bacteria are called outer-membrane vesicles (OMVs) and have a diameter of approximately 20–300 nm. They contain lipids, lipopolysaccharides (LPS), outer membrane proteins, and periplasmic components, and they also include some cytoplasmic components [55,56].

Inherited key antigenic components and pathogen-associated molecular patterns (PAMPs) have been found in bEVs, making them usable as vaccine platforms [56,57]. Moreover, OMV vaccines have already shown their effectiveness on the stage of history. The earliest clinical application of OMV vaccines was undertaken in 1987 in Cuba to combat *Neisseria meningitidis* serogroup B (MenB) [58]. In recent years, intense preclinical research on bEV vaccines against *N. gonorrhoea*, *V. cholerae*, *Mycobacterium tuberculosis*, and nontyphoidal *Salmonella* has also been conducted. These vaccines have been shown to induce both cellular and humoral immune responses [57].

In periodontitis, OMVs secreted by Gram-negative bacteria such as *Porphyromonas gingivalis* (*P. gingivalis*) can invade gingival epithelial cells and impact their functions [59]. Experimental evidence has shown that bEVs derived from periodontal pathogens and oral commensal bacterium can activate Toll-like receptor 2 (TLR2) to promote osteoclast differentiation and exert bone resorption effects [60]. Additionally, bEVs are one of the components of dental biofilm, which threatens oral health [61]. Furthermore, OMVs may transport LPS from the early endosomal compartments to the cytosol of macrophages after endocytosis, mediating immune-inflammatory responses [62]. Studies have detailed the pathogenic role of bEVs derived from periodontal pathogens in periodontal homeostasis [63].

*Burkholderia thailandensis* OMVs can inhibit the bactericidal activity of the oral pathogen *S. mutans*, one of the most cariogenic microorganisms, and disrupt pre-formed *S. mutans* biofilm [64]. All these pieces of evidence indicate that bEVs have intricate connections with oral diseases. Although there are relatively few experimental studies on bEVs and dental caries in recent years, looking ahead, bEVs have a bright prospect as a therapeutic tool for caries.

### 2.3. Structure of sEVs

Currently, research on the structure of sEVs mainly focuses on salivary exosomes. Therefore, this section primarily describes the structure of salivary exosomes, whereas the other two types are not discussed in detail here.

Exosomes are secreted lipid vesicles enveloped by a lipid bilayer membrane [28]. They often share a common set of proteins [3], including membrane transport and fusion proteins (e.g., Rab GTPases, Annexins), proteins associated with endosomal sorting complex required for transport (ESCRT) (e.g., Alix and TSG101), heat shock proteins (HSPs), integrins, tetraspanins (CD63, CD9, CD81), and major histocompatibility complex (MHC) [20,40,65]. In addition, the membrane of exosomes is enriched with sphingolipids, cholesterol, phosphatidylserine (PS), and disaturated lipids [66,67]. The enrichment of sphingolipids and disaturated lipids enhances the rigidity of the membrane [2]. Lipid molecules such as phosphatidylethanolamine (PE), phosphatidylserine (PS), lysophosphatidic acid, and phosphatidic acid (PA) play important roles in maintaining membrane curvature. Moreover, ceramide also contributes to exosome biogenesis [68]. Once secreted to the extracellular environment from parental cells, exosomes will carry nucleic acid, including mRNA and non-coding RNA (small nuclear RNA, miRNA, tRNA) [69]. Equally, exosomal DNA also exists, such as single-stranded DNA, double-stranded DNA [70,71], and mitochondrial DNA [72]. Research has revealed that most DNA contained in tumor-associated exosomes is double-stranded DNA, which may serve as a novel biomarker in the early detection of cancer and metastasis [71].

**Figure 3:**
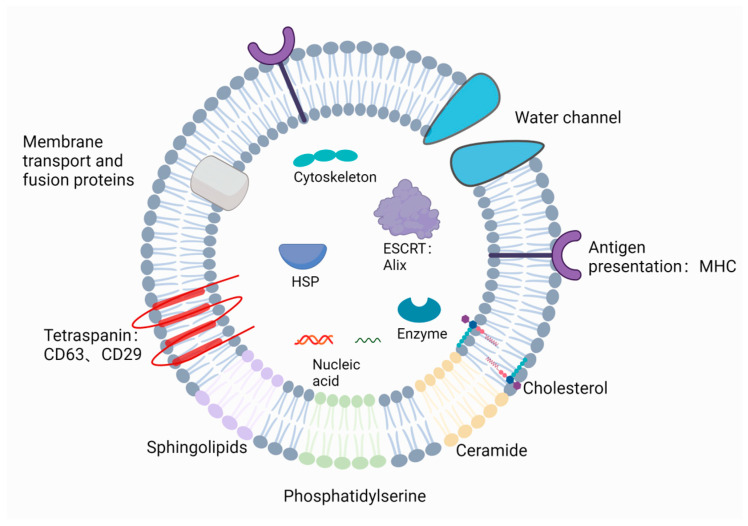
Structure of salivary exosomes. The figure was created with BioRender.com (Accessed on 17 October 2023).

Salivary exosomes, similar to those derived from other bodily fluids, are small vesicles with a diameter of 30–150 nm, surrounded by a phospholipid bilayer membrane [22]. Similarly, salivary exosomes also contain exosome marker proteins, including tetraspanins, HSP, MHC (e.g., MHC class I and MHC class II), membrane transport and fusion proteins, and ESCRT-associated proteins [20]. Cytoskeletal, metabolic, and carrier proteins can also be found in salivary exosomes [20]. In particular, salivary exosomes are typically rich in aquaporins (apical plasma membrane channels), reflecting the unique features of exosomes secreted by salivary glands [20,22,50]. Of note, salivary exosomes contain tissue factor (TF), which can trigger factor-VII-mediated coagulation and shorten clotting time [19]. Except for proteins, salivary exosomes also carry nucleic acids from their donor cells [22,51]. The databases Vesiclepedia [73] and ExoCarta [74] have catalogued the nucleic acids, proteins, and lipids currently identified in salivary exosomes. Changes in the nanoscale structure of salivary exosomes are associated with disease. In fact, cancer exosomes have a larger size compared with normal exosomes, and their CD63 surface density also increases [19,20] (Figure 3).

### 2.4. Isolation of sEVs

At present, there is much research being conducted into the isolation of EVs from other biofluids, such as blood and urine. However, there are limited reports on isolating sEVs. Some aspects need to be taken into consideration, such as cells and solid contaminants, and viscosity of samples. Methods used to isolate sEVs mainly consist of ultracentrifugation (UC), magnetic-bead-based exosome extraction, and density gradient ultracentrifugation.

The most popular method for isolating salivary exosomes is UC, which can yield exosomes with minimally contaminated pellets. However, the process is time-consuming and it requires an expensive and bulky device [75]. In addition, high viscosity with saliva requires a longer centrifugation period, thus diminishing the exosomes’ integrity [76]. A study on extracting salivary exosomes using the magnetic bead affinity method has been conducted [53]. However, the relevant research is limited, and effort is required before the magnetic bead affinity method is used in clinical settings. Density gradient ultracentrifugation is a method of EV fractionation based on density. The viscosity of EVs prepared from saliva is higher than that of EVs from blood, and, thus, the protocol must be modified. A pretreatment step and iodixanol are required to concentrate the EVs to a 1.1 g/mL density [77].

Alongside the traditional methods, new technology has also been established. For instance, ExoQuick (EQ), a proprietary reagent, has been used for isolating exosomes from saliva. Pellets obtained by EQ have larger diameters, which are readily discernable and convenient to manipulate compared with UC. The main advantages of EQ consist of technical simplicity, quick isolation of exosomes, and the ability to operate with a small sample volume. However, EQ pellets also contain higher biological impurities [75]. RNAPro•SAL™ (Oasis Diagnostics^®^ Corporation, Vancouver, WA, USA), an oral specimen collection system, can collect controlled and standardized oral fluid specimens. RNAPro•SAL™ used in conjunction with EQ can simplify exosome isolation, with less non-exosomal contaminating materials and without exosomes loss [78].

These interesting findings suggest that sEVs can serve as biomarkers for diseases and have potential applications in disease diagnosis and monitoring. However, there remain challenges in the isolation of EVs from saliva. Salivary components and pH value can change in accordance with circadian rhythms [79,80]. Thus, sample collection at different time points may result in variability in sEVs. Additionally, the viscosity of saliva not only makes the processing more complicated but also compromises the purity of saliva exosomes obtained through UC [76]. Each method mentioned above has limits, and, therefore, a uniform purification approach for sEVs is lacking. Consequently, endeavors are required to develop an efficient, reproducible, standardized isolation method.

## 3. Roles of sEVs in Human Diseases

### 3.1. Biomarkers

Biomarkers can provide useful information for disease prevention, diagnosis, prognosis, and treatment through measurement or identification in vitro [81]. In addition, biomarkers must be accurately and reliably measured within a defined context of application [82,83].

In saliva, EVs protect cargoes from contamination of the oral environment, thereby stabilizing components such as miRNA and enhancing the accuracy of detection [84]. Additionally, saliva is non-coagulating and easily collected in a non-invasive manner, resulting in good patient compliance [12,15]. Numerous studies have highlighted the potential of molecules in sEVs as promising biomarkers for various diseases.

#### 3.1.1. Oral Diseases

Ninety percent of the global population will experience oral diseases during their lifetime, primarily including dental caries, periodontal diseases, and oral cancer [85]. Dental caries [86] and periodontal diseases [87] can both lead to tooth loss, impacting oral health and overall health through reduced mastication function. Oral cancer (OC) is the sixth most common cancer worldwide [88], with a high mortality rate and an increasing trend in recent years, posing a serious threat to human health [89]. However, the prevalence, severity, and often overlooked nature of oral diseases [90] make them one of the most common global public health issues [85]. Therefore, emphasizing oral health and the treatment of oral diseases is of the utmost importance. The utilization of sEVs as biomarkers can contribute to the diagnosis, prognosis assessment, and evaluation of treatment efficacy for oral diseases (Table 1), thereby promoting oral and overall health.

##### Oral Cancers

OC can occur in the lips, tongue, gums, and lining of the oral cavity [12]. In total, 90% are derived from squamous cells, and are known as oral squamous cell carcinoma (OSCC) [83,88]. One of the main reasons for the high mortality of OC is delayed diagnosis, emphasizing the importance of screening and early diagnosis [88,91]. In recent years, there have been many studies on sEVs as biomarkers of OC.

Proteomic analysis of sEVs has revealed differences between OSCC patients and healthy individuals [92,93]. These differentially expressed proteins are mainly associated with immune responses, cell growth and proliferation, and inflammatory responses, all of which are closely related to the development of cancer [93]. The differential expression of these proteins may partly reflect biological changes in patients compared with healthy individuals, and therefore have potential as OSCC biomarkers [92,93]. Compared with healthy individuals, in OSCC patients, CD63 was highly expressed, whereas CD81 and CD9 were lowly expressed [94,95]. Alix (apoptosis linked gene-2-interacting protein X) was highly expressed the in sEVs of OC patients, and its receiver operating curve (ROC) showed good diagnostic performance, indicating it as a promising biomarker [96]. In addition, Sun et al. tried to screen out biomarkers for evaluating the therapeutic effect of OSCC. What is interesting is that they invented a new method for isolating EVs—bi-functionalized magnetic beads (BiMBs), which were shown to effectively isolate EVs from a small amount of saliva. After analyzing the proteomic profile of sEVs, among the numerous upregulated proteins, three whole proteins and three phosphoproteins were found to have sensitive responses to surgical treatment of OSSC, with the potential to evaluate the therapeutic effect [97].

MiRNAs in sEVs are also promising biomarkers for OC, including *miR-1307-5p*, *miR-24-3p*, *miR-10b-5p*, *miR-486-5p*, and so on (Table 1) [98,99,100]. Among them, *miR-1307-5p* could promote OSCC progression, and the overexpression of it suggested a poor prognosis for OSCC patients [98]. A review assessed the quality of the literature published between 2010 and 2021 to identify the most promising biomarkers for oral and oropharyngeal cancer. The authors identified *miR-10b-5p*, *miR-486-5p*, *miR-24-3p*, and *miR-200a* as having significant clinical utility as biomarkers [101]. Interestingly, Patel et al. combined *miR-140-5p*, *miR-143-5p*, and *miR-145-5p*, which were significantly downregulated in OSCC patients, and the combined 3-RNA showed good diagnostic performance for OSCC. This provides a concept for the seeking of new useful biomarkers—combining several differentially expressed substances to improve diagnostic performance [102].

It is worth noting that some miRNAs are not only related to OSCC, but also to other cancers. *MiR-24-3p* has been shown to play a role in promoting the progression of breast cancer [103], bladder cancer [104], hepatocellular carcinoma [105], and Hodgkin lymphoma [99,106]. *MiR-140* has been reported to exhibit decreased expression in various cancers, including breast cancer [107], prostate cancer [108], lung cancer [109], and so on. Furthermore, *miR-140*, *miR-143*, and *miR-145* have been shown to inhibit the progression of gastric cancer [102,110,111]. This poses challenges for their use as OSCC biomarkers, as the differential expression of these miRNAs may be due to other cancers. If differentially expressed molecules unique to OSCC can be found, it will make a huge contribution to the application of oral cancer biomarkers.

##### Periodontal Disease

Periodontal disease is a common inflammatory condition in the oral cavity that can lead to tooth loss [87]. Additionally, periodontal disease is associated with various inflammation-related diseases, including diabetes, cardiovascular diseases, neurodegenerative diseases, autoimmune diseases, and cancer. Treatment of periodontitis may help to improve these associated comorbidities [112]. Maintaining periodontal health not only benefits oral health but also plays a crucial role in overall well-being. Recent studies have demonstrated the potential of sEVs in the diagnosis and detection of periodontal diseases.

The proteomics of sEVs in periodontitis patients is different from that in healthy individuals [113,114]. As with OSCC, CD9 and CD81 were lowly expressed in sEVs from patients with periodontitis [113]. Conversely, immune-related proteins such as complement components were highly expressed, and thus have the potential to serve as biomarkers [114].

It was reported that many RNAs in sEVs were potential biomarkers for periodontitis. A comparative study profiled eight salivary exosomal miRNA samples, identifying 1995 differentially expressed miRNAs in chronic periodontitis (CP) saliva compared with healthy controls. A promising biomarker for further investigation was *hsa-miR-125a-3p* which had a strong correlation with periodontal pocket depth (PPD) [115]. Additionally, a study found that *miR-223-3p* was downregulated in periodontitis patients, and, thus, it could serve as a biomarker for diagnosing and assessing disease severity [21]. Another study found that the level of PD-L1 mRNA in sEVs showed differences at different stages of periodontitis, indicating that detection of the PD-L1 mRNA could also provide information on the severity of periodontitis [116]. In addition, in a two-year cohort study, several salivary miRNAs, including *hsa-miR-5571-5p*, *hsa-let-7f-5p*, *hsa-miR-99a-5p*, *hsa-miR-28-5p*, and *hsa-miR-320d*, showed a fair discrimination power for periodontitis progression. These miRNAs could serve as biomarkers for monitoring the progression of periodontitis, contributing to precision medicine for this disease [117]. A pilot study discovered that specific miRNAs (*hsa-miR-140-5p*, *hsa-miR-628-5p*, and *hsa-miR-146a-5p*) exhibited a significant increase in sEVs from periodontitis patients, demonstrating a high diagnostic accuracy when considering all three miRNAs together (AUC of ROC = 0.96) [118]. Furthermore, Global 5mC hypermethylation in sEVs effectively distinguishes periodontitis patients from both gingivitis patients and healthy individuals, indicating its potential as a biomarker for periodontitis [119].

##### Primary Sjögren’s Syndrome

Primary Sjögren’s syndrome (pSS) is a multifactorial autoimmune disease with a triad of dryness, pain, and fatigue [120,121]. Aqrawi et al. characterized the proteomics of sEVs in patients with pSS and healthy individuals and identified the top five upregulated proteins, as listed in Table 1. These proteins are promising biomarkers for pSS and may be used for prognosis evaluation and disease monitoring [122].

Another study found that 121 proteins in sEVs were differentially expressed in pSS patients compared with healthy controls. The most differentially expressed proteins were related to immune response, including the S100 protein family (S100A7, A9, A12), resistin (RETN), and so on. Further studies are needed to identify the promising biomarkers among those proteins [123].

##### Oral Lichen Planus

Oral lichen planus (OLP), most common in the buccal mucosa, is an immune-mediated inflammatory disease. It can be asymptomatic or painful [124]. Byun et al. compared miRNA profiles in sEVs between OLP patients and healthy controls and found that *miR-4484* was significantly upregulated in OLP patients, suggesting that it could be used as a potential biomarker for OLP [125].

**Table 1:** Potential biomarkers of oral diseases in sEVs.

Disease	Type	Potential Biomarker	Expression	Application	AUC of ROC Curve	Ref.	Year
OC	Protein	CD81 and CD9	Downregulated	Diagnosis	/	[94,95]	2011; 2016
		CD63	Upregulated	Diagnosis	/	[94]	2016
		Alix	Upregulated	Diagnosis	AUC = 0.712	[96]	2021
		Three full proteins (HEP2, NHERF-2, and MMP25) and three phosphoproteins (PGM 1, ACLY, and KPCD)	Upregulated	Assessing OSCC therapeutical outcomes	/	[97]	2023
OC	RNA	*miR-1307-5p*	Upregulated	Diagnosis and suggesting poor prognosis	/	[98]	2022
		*miR-24-3p*	Upregulated	Diagnosis	AUC = 0.738, *p* = 0.02	[99]	2020
		*miR-486-5p*	Upregulated	Diagnosis	AUC = 0.67, *p* = 0.05	[100]	2022
		*miR-10b-5p*	Downregulated	Diagnosis	AUC = 0.58, *p* = 0.33	[100]	2022
		*miR-140-5p*, *miR-143-5p* and *miR-145-5p*	Downregulated	Diagnosis	(Combination of these three RNAs) AUC of 0.93 (*p* < 0.0001)	[102]	2023
Periodontal disease	Protein	CD9, CD81	Downregulated	Diagnosis	/	[113]	2019
		Complement components and chemokine ligand 28	Upregulated	Diagnosis	/	[114]	2020
	RNA	*hsa-miR-125a-3p*	Downregulated	Diagnosis of CP	AUC = 1, *p* = 0.02	[115]	2020
		*miR-223-3p*	Downregulated	Diagnosis and assessing its severity	/	[21]	2021
		PD-L1 mRNA	Upregulated	Diagnosis and assessing its severity	/	[116]	2019
Periodontal disease	RNA	*hsa-miR-5571-5p*	Upregulated	Monitoring and diagnosing the progression of periodontitis	AUC = 0.849, *p* < 0.001	[117]	2021
		*hsa-let-7f-5p*	Downregulated		AUC = 0.705, *p* = 0.02		
		*hsa-miR-99a-5p*	Downregulated		AUC = 0.747, *p* = 0.0054		
		*hsa-miR-28-5p*	Downregulated		AUC = 0.711, *p* = 0.017		
		*hsa-miR-320d*	Downregulated		AUC = 0.705, *p* = 0.02		
		*hsa-miR-140-5p*, *hsa-miR-628-5p*, and *hsa-miR-146a-5p*	Upregulated	(Panel of three miRNAs) diagnosis of periodontal disease status	AUC = 0.96, *p* < 0.0001 for periodontitis; AUC = 0.78, *p* = 0.0006 for gingivitis	[118]	2020
	/	Global 5mC hypermethylation	Significantly increased	Diagnosis of periodontitis	AUC = 1, *p* = 0.001	[119]	2021
PSS	Protein	APMAP, GNA13, WDR1, SIRPA, LSP1	Upregulated	Diagnosis and prognosis evaluation and disease monitoring	/	[122]	2017
		S100A7, S100A8, S100A9, S100A11, and S100A12, RETN, SERPINB1 and SERPINB5, AZU1, CD14, ANXA2, CFL-1, LCP1, MIF	Upregulated	Diagnosis	/	[123]	2021
OLP	RNA	*miR-4484*	Upregulated	Diagnosis	/	[125]	2015

Abbreviations. ACLY: adenosine triphosphate citrate lyase; Alix: apoptosis linked gene-2-interacting protein X; ANXA2: annexin A2; APMAP: adipocyte plasma membrane-associated protein; AZU1: azurocidin; AUC: area under curve; CFL-1: cofilin-1; CP: chronic periodontitis; GNA13: guanine nucleotide-binding protein subunit alpha-13; HEP2: heparin cofactor 2; KPCD: protein kinase C delta type; LCP1: plastin-2; LSP1: lymphocyte-specific protein 1; MIF: macrophage migration inhibitory factor; MMP25: matrix metalloproteinase-25; NHERF-2: Na+/H+ exchanger regulatory factor 2; OC: oral cancer; OLP: oral lichen planus; OSCC: oral squamous cell carcinoma; PD-L1: programmed death-ligand 1; pSS: primary Sjögren’s syndrome; PGM 1: phosphoglucomutase 1; Ref: reference; RETN: resistin; ROC: receiver operating curve; SEVs: saliva extracellular vesicles; SERPINB1 and SERPINB5: serpin peptidase inhibitors; SIRPA: tyrosine-protein phosphatase nonreceptor type substrate 1; S100A7, S100A8, S100A9, S100A11, and S100A12: S100 protein family; WDR1: WD repeat-containing protein 1.

#### 3.1.2. Non-Oral Diseases

SEVs are not only associated with oral diseases but also with non-oral diseases [126,127,128]. Research indicates that sEVs can serve as potential biomarkers for numerous non-oral diseases (Table 2).

##### Lung Cancer

Lung cancer is one of the most common cancers globally and stands as a primary contributor to cancer-related fatalities [129]. Due to the lack of advanced diagnostic methods, 75% of patients are diagnosed in an advanced stage [130]. Recently, sEVs have gained much attention as potential early diagnostic tools.

Wahid et al. used the Ti4+- IMAC (immobilized metal affinity chromatography) method to compare the phosphoprotein profiles in the sEVs of lung cancer patients and normal individuals. A total of 857 unique phosphopeptides corresponding to 721 saliva phosphoproteins were identified. Among the identified phosphorylation sites, 37 sites and 217 sites were, respectively, upregulated and downregulated in patients, holding potential for the early detection of lung cancer via salivary diagnostics [131].

*MiRNA-205* has been identified as a potential biomarker for non-small-cell lung cancer [132]. Based on a high sensitivity for *miRNA-205*, an inexpensive, user-friendly, and highly sensitive lung cancer diagnostic kit (LCDK) has been developed. The LCDK enables rapid and non-invasive lung cancer diagnosis using clinical samples of saliva and urine within a short timeframe [133]. The invention of this diagnostic kit shows a significant advancement in the clinical application of sEVs as biomarkers, but its widespread clinical adoption still requires much effort.

##### Other Cancers

Aside from lung cancer, other cancers, including esophageal, pancreaticobiliary, and prostate cancer, may be diagnosed by detecting sEVs.

Lin et al. suggested that salivary exosomal GOLM1 (Golgi membrane protein 1)-NAA35 (Nα-acetyltransferase 35 gene) chimeric RNA (seG-NchiRNA) was an efficient candidate non-invasive biomarker for esophageal cancer, which could be conveniently and reliably used to evaluate treatment outcomes and risk of recurrence, and for early detection [134]. In addition, a signature based on salivary exosomal tsRNAs exhibited diagnostic and prognostic potential for esophageal cancer, as well as the potential to guide treatment, which may contribute to precision medicine for esophageal cancer [135].

In a study of pancreaticobiliary carcinoma, it was found that *miR-1246* and *miR-4644* in the salivary exosomes of patients were significantly higher than in those from healthy controls, and the combination of the two showed a good disease diagnosis ability [136].

In addition, a study found that *hsa-mir-331-3p* and *hsa-mir-200b* in prostate cancer were significantly downregulated compared with the control group, suggesting that they can be used as potential biomarkers for prostate cancer [137].

##### Neurodegenerative Diseases

Neurodegenerative diseases are the gradual loss of selective and fragile neuronal populations. The clinical manifestations are cognitive and behavioral disorders, including Alzheimer’s disease (AD), Parkinson’s syndrome and motor neuron disease [138].

A study presented an innovative approach utilizing nanoparticle tracking analysis (NTA) for the direct quantification of salivary exosome concentration and its association with the advancement of cognitive decline in AD. It showed a negative correlation between exosome concentration and Addenbrooke’s cognitive examination III (ACE III) scores. A lower score on the ACE III indicates a more severe condition in patients with AD. It suggested that employing the nano-tracking technique to measure salivary exosome concentration holds promise as a screening method for early disease detection [139].

Rani et al. found that the abundance of neuron-derived salivary exosomes and the level of phosphorylated α-synuclein (α-syn) in salivary exosomes in patients with Parkinson’s disease (PD) were significantly higher than those in healthy controls [140]. Similarly, Cao et al. collected saliva samples from PD patients and healthy controls to detect the level of α-syn in the salivary exosomes of each group. It was found that the levels of α-syn_Olig_ (oligomeric α-syn) and α-syn_Olig_/α-syn_Total_ (Total α-syn) in the salivary exosomes of PD patients were higher than in those of healthy controls, which could be used to distinguish PD and healthy people [141]. These findings suggested that the detection of α-syn in sEVs might be helpful for the diagnosis of PD.

Amyotrophic lateral sclerosis (ALS), alternatively referred to as motor neuron disease, is a fatal neurodegenerative disorder affecting the central nervous system [142,143]. Sjoqvist et al. established a workflow for the analysis of saliva and sEVs to compare protein profiles between ALS patients and healthy individuals. The study revealed a downregulation trend in ZNF428 (zinc finger protein 428) in sEVs from ALS patients compared with the control group [144].

##### Traumatic Brain Injury

Traumatic brain injury (TBI) often results in mortality and acquired disability among people of all ages, including both adults and children [145]. According to the severity of clinical symptoms, TBI can be divided into mild, moderate, and severe. Secondary damage is caused by cellular and molecular mechanisms that respond to initial damage and may last for a long time [146]. There is a need for objective and quantifiable biomarkers to aid in the diagnosis of acute TBI and predict the risk of long-term consequences.

A study recruited healthy individuals, concussion patients, and TBI patients in the emergency department and performed real-time PCR analysis on their sEVs. Compared with the control group, 57 genes and 56 genes were upregulated in emergency department patients and patients with concussion, respectively. Additionally, three genes, namely, *CDC2*, *CSNK1A1*, and *CTSD*, were upregulated in both emergency department patients and patients with concussion. This indicates that genes within sEVs may serve as potential biomarkers for mild TBI [145]. Similarly, Cheng Y. et al. found that a total of nine genes were upregulated in emergency department patients’ sEVs compared with healthy controls, and a total of 13 genes were upregulated in patients with concussion. Every group displayed its own unique profile. This indicates that sEVs have the potential to be applied in the diagnosis and assessment of the severity of TBI [147].

##### Mental Disorders

Mental disorders have attracted attention due to their high incidence in recent years. Some researchers have selected potential biomarkers by analyzing the proteomics of sEVs [148,149]. In a study, 10 candidate proteins related to emotional disorders were identified in sEVs, and it was found that the level of neuronal glycoprotein M6a (GPM6a) was positively correlated with the stress level in depressed individuals. Additionally, there was a difference in GPM6a levels between treated depression patients and the untreated group. This indicates that GPM6a can be used as a potential biomarker for stress and has a potential therapeutic monitoring effect [149]. In addition, studies have shown that phosphoglycerate kinase 1 (PGK1) in sEVs is a potential biomarker for assessing fatigue levels [150].

**Table 2:** Potential biomarkers of non-oral diseases in sEVs.

Disease			Type	Potential Biomarker	Expression	Application	AUC of ROC Curve	Ref.	Year
Lung cancer			Protein	Differentially expressed phosphopeptides	37 phosphopeptides were upregulated	Diagnosis	/	[131]	2022
				Differentially expressed phosphopeptides	217 phosphopeptides were downregulated	Diagnosis			
Other cancers	Esophageal cancer		RNA	seG-NchiRNA	Upregulated	Diagnosis and evaluating treatment response and risk of recurrence	AUC = 0.912 *p* < 0.001	[134]	2019
Other cancers	Esophageal cancer		RNA	A combination of a tsRNA (*tRNA-GlyGCC-5*) and a previously undocumented small RNA	Upregulated	Diagnosis and prognosis and guiding therapy	/	[135]	2022
	Pancreaticobiliary carcinoma		RNA	*miR-1246* and *miR-4644*	Upregulated	Diagnosis	The combination of these two RNAs showed AUC = 0.833 *p* = 0.005	[136]	2016
	Prostate cancer		RNA	*miR-331-3p* and *miR-200b*	Downregulated	Diagnosis	AUC = 0.663 (*miR-200b*); AUC = 0.648 (*miR-331-3p*)	[137]	2022
Neurodegenerative diseases	PD		Protein	α-synuclein	Upregulated	Diagnosis	/	[140]	2019
Neurodegenerative diseases		PD	Protein	α-synOlig	Upregulated	Diagnosis	AUC = 0.941	[141]	2019
		ALS	Protein	ZNF428	Downregulated	Diagnosis	/	[144]	2023
TBI			Gene	*CDC2*, CSNK1A1 and CTSD	Upregulated	Diagnosis of mild TBI	/	[145]	2019
				*ALOX5, ANXA3, CASP1, IL2RG, ITGAM, ITGB2, LTA4H, MAPK14,* and *TNFRSF1A*	Upregulated	Diagnosis and assessing the severity of TBI	/	[147]	2020
Mental disorders			Protein	GPM6a	Upregulated	Diagnosis of stress and monitoring therapeutic effect	/	[149]	2020
			Gene	*PGK1*	Upregulated	Assessing fatigue levels	/	[150]	2022

Abbreviations. AD: Alzheimer’s disease; *ALOX5*: *Arachidonate 5-Lipoxygenase*; ALS: amyotrophic lateral sclerosis; *ANXA3*: *annexin A3*; AUC: area under curve; *CASP1*: *caspase-1*; *CDC2*: *cell division cycle 2*; *CSNK1A1*: *Casein Kinase 1 Alpha 1*; *CTSD*: *cathepsin D*; GPM6a: glycoprotein M6a; *IL2RG*: *interleukin-2 receptor subunit gamma*; *ITGAM*: *integrin alpha M*; *ITGB2*: *integrin beta-2 (CD18)*; *LTA4H*: *leukotriene A4 hydrolase*; *MAPK14*: *mitogen-activated protein kinase 14* (*p38*); PD: Parkinson’s disease; *PGK1*: *phosphoglycerate kinase 1; Ref: reference*; ROC: receiver operating curve; seG-NchiRNA: salivary exosomal GOLM1 (Golgi membrane protein 1)-NAA35 (Nα-acetyltransferase 35 gene) chimeric RNA; SEVs: saliva extracellular vesicles; TBI: traumatic brain injury; *TNFRSF1A*: *tumor necrosis factor receptor superfamily member 1A* (*TNF receptor 1*); ZNF428: zinc finger protein 428.

#### 3.1.3. Physiological Condition: Age

In addition to biomarkers of diseases, sEVs can also reflect physiological conditions such as age. Differential protein expression was observed in salivary exosomes between menopausal and adolescent women [151]. Menopausal women exhibited a higher total protein content in their salivary exosomes compared with adolescent women, especially major salivary proteins such as immunoglobulins and amylase. In contrast, adolescent women had greater protein diversity than menopausal women. The levels of proteins related to ribosomes and structural molecules were higher in adolescent women. This indicates that sEV proteomics has the potential to provide age information.

### 3.2. Non-Biomarker Functions

SEVs have various functions beyond their potential use as biomarkers.

#### 3.2.1. Innate Immune Defense

The Zika virus (ZIKV) is transmitted mainly by mosquitoes, and human-to-human transmission also exists, but no record of saliva transmission has been found. Studies found that sEVs prevented the attachment and infection of ZIKV at a natural concentration in saliva, suggesting that sEVs might have an innate immune defense effect [24].

#### 3.2.2. Hemostasis

A study found that sEVs exposed P-selectin ligand (CD24) and coagulation TF. Thus, sEVs could bind to P-selectin to act on activated platelets and promote hemostasis at the site of skin injury [152].

#### 3.2.3. Therapy

An animal study found that the injection of salivary exosomes into diabetic rats could reduce blood glucose levels, and improved salivary gland function, suggesting that salivary exosomes might be a new treatment for diabetes-associated xerostomia and salivary gland dysfunction [153].

In addition, it was identified that sEVs have potential therapeutic effects in corneal wound healing. In vitro, primary corneal stromal cells were treated with salivary exosomes, and scratches and cell migration were measured at various time points after stimulation. It was found that compared with the control group, the cleavage of the corrugated protein increased after treatment with salivary exosomes, which promoted wound healing and cell migration [154].

#### 3.2.4. Carrier for Drug Delivery Systems

Currently, drug delivery systems (DDSs) have attracted much attention in cancer treatment due to their significant reduction in toxicity and adverse reactions [155]. As carriers, exosomes have the advantages of good biocompatibility, low immunogenicity, the ability to cross various biological barriers, and the modifiability of membrane surface molecules [156]. Various types of exosomes, including plasma exosomes, tumor-cell-derived exosomes, and mesenchymal-stem-cell-derived exosomes have been investigated for possible applications as carriers for DDSs [26,157,158], but there is still a lack of research on salivary exosomes as carriers. Future studies may explore the possibility of salivary exosomes as drug delivery systems.

## 4. Conclusions

SEVs have been widely studied as biomarkers of oral and systemic diseases, including OC, periodontitis, lung cancer, neurodegenerative diseases, traumatic brain injury, etc. Most studies determine a component in sEVs as a potential biomarker based on its difference in expression between patients and healthy controls. Interestingly, recent studies have often combined several differentially expressed components in a disease to form a combined biomarker to improve the precision of disease diagnosis [97,102]. However, most studies have focused only on screening differentially expressed components. There is still a gap between scientific research and the application of sEVs as biomarkers in clinical practice. Taking OSCC as an example, the gold standard for its diagnosis is still tissue biopsy [91]. Most research on sEVs as biomarkers is still in the laboratory stage, due to the need for complex and expensive separation, characterization, and analysis techniques [88]. Surprisingly, an inexpensive, user-friendly, and highly sensitive diagnostic kit for lung cancer has been developed [133]. Although it has not been widely used in clinical practice, this development suggests that it is feasible to use sEVs as biomarkers in clinical practice. Furthermore, it is also necessary to consider whether it is beneficial to use sEVs as biomarkers. For some diseases that are easy to diagnose and for which it is easy to evaluate the effect of treatment according to symptoms, it is necessary to consider whether the previous diagnosis method is more appropriate and beneficial.

In addition, sEVs also have immune defense, hemostatic, and therapeutic properties. Furthermore, they have great application value as carriers in DDSs. Future research should focus on the therapeutic roles of sEVs and their potential as carriers in DDSs.

In summary, non-invasive and easy-to-obtain sEVs have promising prospects for application as biomarkers and can bring great benefits to patients. The therapeutic effects of sEVs and their role as a carrier require further study.

## Data Availability

Not applicable.
